# Protection of Radiation-Induced Damage to the Hematopoietic System, Small Intestine and Salivary Glands in Rats by JNJ7777120 Compound, a Histamine H_4_ Ligand

**DOI:** 10.1371/journal.pone.0069106

**Published:** 2013-07-26

**Authors:** Diego J. Martinel Lamas, Eliana Carabajal, Juan P. Prestifilippo, Luis Rossi, Juan C. Elverdin, Susana Merani, Rosa M. Bergoc, Elena S. Rivera, Vanina A. Medina

**Affiliations:** 1 Laboratory of Radioisotopes, School of Pharmacy and Biochemistry, University of Buenos Aires, Buenos Aires, Argentina; 2 Patagonic National Center-CONICET, Chubut, Argentina; 3 Physiology Department, School of Dentistry, University of Buenos Aires, Buenos Aires, Argentina; 4 Center of Reproduction Research, School of Medicine, University of Buenos Aires, Buenos Aires, Argentina; 5 National Scientific and Technical Research Council (CONICET), Buenos Aires, Argentina; University of Science and Technology of China, China

## Abstract

Based on previous data on the histamine radioprotective effect on highly radiosensitive tissues, in the present work we aimed at investigating the radioprotective potential of the H_4_R ligand, JNJ7777120, on ionizing radiation-induced injury and genotoxic damage in small intestine, salivary glands and hematopoietic tissue. For that purpose, rats were divided into 4 groups. JNJ7777120 and JNJ7777120-irradiated groups received a daily subcutaneous JNJ7777120 injection (10 mg/kg) starting 24 h before irradiation. Irradiated groups received a single dose of 5 Gy on whole-body using Cesium-137 source and were sacrificed 3 or 30 days after irradiation. Tissues were removed, fixed, stained with hematoxylin and eosin or PAS staining and histological characteristics were evaluated. Proliferative and apoptotic markers were studied by immunohistochemistry, while micronucleus assay was performed to evaluate DNA damage. Submandibular gland (SMG) function was evaluated by methacholine-induced salivation. Results indicate that JNJ7777120 treatment diminished mucosal atrophy and preserved villi and the number of crypts after radiation exposure (240±8 vs. 165±10, P<0.01). This effect was associated to a reduced apoptosis and DNA damage in intestinal crypts. JNJ7777120 reduced radiation-induced aplasia, preserving medullar components and reducing formation of micronucleus and also it accelerated bone marrow repopulation. Furthermore, it reduced micronucleus frequency in peripheral blood (27±8 vs. 149±22, in 1,000 erythrocytes, P<0.01). JNJ7777120 completely reversed radiation-induced reduced salivation, conserving glandular mass with normal histological appearance and reducing apoptosis and atrophy of SMG. JNJ7777120 exhibits radioprotective effects against radiation-induced cytotoxic and genotoxic damages in small intestine, SMG and hematopoietic tissues and, thus, could be of clinical value for patients undergoing radiotherapy.

## Introduction

Effective radiotherapy to treat cancer patients should include maximal tumor cell killing with minimal injury to normal tissue. A cautious balance between the total dose of radiation delivered and the threshold limit of the surrounding normal critical tissues is essential to optimize outcomes. The prevention or treatment of early and late radiotherapy effects would improve quality of life and increase cancer curability by intensifying therapies. In this sense, the role of radioprotective compounds is of utmost importance in clinical radiotherapy [1,2]

Several compounds have been reported to offer potential for radiation protection, but most of them are not suitable for clinical application due to toxicity and poor specificity [1–3]. Ionizing radiation-induced free radicals and reactive oxygen species (ROS) damage vital cellular targets such as DNA and membranes, which could lead to cell death and functional failure of irradiation-sensitive tissues such as gastrointestinal epithelium and hematopoietic system, in which the response is determined by a hierarchical cell lineage, composed of stem cells and their differentiating offspring [1,2].

Bone marrow is the primary hematopoietic tissue in mammals producing all blood cells and whole-body irradiation reduced bone marrow cellularity and blood cell count [1,4]. Within the digestive system small intestine and salivary glands are highly radiosensitive [1]. Side effects of pelvic or abdominal radiation therapy include nausea, diarrhea, and villous atrophy while radiation-induced xerostomia still represents a common adverse effect after radiotherapy for head-and-neck malignancies [1–3].

In this regard, it was previously reported that histamine significantly protects in mice and rats two of the most radiosensitive tissues, small intestine and bone marrow, from high doses of gamma radiation [5–7]. In agreement with these data, histamine prevents functional and histological alterations of salivary glands produced by ionizing radiation [3]. In addition, histamine has the ability to enhance the radiosentivity of breast malignant cells [8].

Histamine [2-(4-imidazolyl)-ethylamine] is an endogenous biogenic amine and is known since long to be a pleiotropic mediator in different (patho) physiological conditions, which exerts its actions through the interaction with four histamine receptor subtypes [9]. The discovery of the human histamine H_4_ receptor (H_4_R) has revealed novel functions for histamine and opening new perspectives in histamine pharmacology research. H_4_R appeared to have a selective expression pattern restricted to medullary and peripheral hematopoietic cells and it is assumed to play an important pro-inflammatory role in various diseases, including bronchial asthma, atopic dermatitis, and pruritus. In addition, H_4_R was reported to be present on other cell types including intestinal epithelium, spleen, lung, stomach, central nervous system, salivary glands and cancer cells [9–11]. Over the past decade, the indole derivative JNJ7777120 (Table 1), developed by Johnson and Johnson Pharmaceuticals, has more than a thousand-fold selective over other histamine receptor subtypes and has become the reference H_4_R antagonist, being extensively used to assess the H_4_R pathophysiological role [10,12,13].

**Table 1:** Molecular characteristics of JNJ7777120.

**IUPAC**	1-[(5-Chloro-1*H*-indol-2-yl)carbonyl-]-4-methylpiperazine
**Molecular Weight**	277.76
**Source**	Johnson & Johnson Pharmaceutical Research and Development, San Diego, CA

Based on previous data on the histamine radioprotective effect on highly radiosensitive tissues, in the present work we aimed to investigate the radioprotective potential of the H_4_R ligand, JNJ7777120, evaluating its effect on reducing ionizing radiation-induced injury and genotoxic damage in the rat small intestine, salivary glands and hematopoietic tissue.

## Materials and Methods

### Treatment and irradiation

Eighty 10-week-old male Sprague-Dawley rats, weighing 200-230 g were purchased from the Division of Laboratory Animal Production, School of Veterinary Sciences, University of La Plata, Buenos Aires and were randomly separated into 4 groups (n = 20 each). Rats were maintained in our animal health care facility at 22 to 24°C and 50% to 60% humidity on a 12 h light/dark cycle with food and water available *ad libitum*. To determine the potential radioprotective effect of JNJ7777120 (Table 1) on small intestine, SMG and bone marrow we used the same experimental procedures that were previously described in detail [3,5–7]. Briefly, JNJ7777120 and JNJ7777120-5Gy groups received a daily subcutaneous JNJ7777120 injection (10 mg/kg) starting 24 h before irradiation and which continued until 3 days post-irradiation while untreated groups received saline. Irradiated groups received a single dose of 5 Gy on whole-body using Cesium-137 source and animals were sacrificed 3 or 30 days after irradiation.

Three days post-irradiation methacholine-induced salivary secretion was measured (n = 4 each group) or animals were anesthetized by CO_2_ inhalation and then immediately sacrificed by cervical dislocation and SMG, small intestine and femur bone marrow were removed, weighed, and histological and histochemical characteristics were evaluated (n = 6 each group). Additionally, 30 days post-irradiation other group of animals (n = 6 each group) were sacrificed to evaluate histological parameters. For oxidative damage evaluation 3 days post-irradiation additional animals were employed (n = 4 each group).

Animal procedures were in accordance with recommendations from the Guide for the Care and Use of Laboratory Animals of the National Research Council, USA, 1996, 2011, and protocols were approved by the Ethical and Educational Committee for the Use and Care of Laboratory Animals of the School of Pharmacy and Biochemistry.

### Histopathological studies

SMG and small intestine were removed and were fixed with 10% neutral buffered formalin while femur bone marrows were fixed with Bouin’s solution. Tissue samples were embedded in paraffin and cut into serial sections of 4 µm thick. The histopathological characteristics were examined on tissue sections after hematoxylin-eosin (H&E) staining as it was previously described [3,6,7]. Also, specimens were stained with periodic acid schiff (PAS) staining and counterstained with hematoxylin to evaluate glycogen deposits.

### Immunohistochemical staining

Immunohistochemistry was performed as it was previously described [3,6,7]. Briefly, after blocking tissues were incubated with primary mouse anti-proliferating cell nuclear antigen (PCNA, 1:100, Dako Cytomation, Glostrup, Denmark), rabbit anti-aquaporin 5 (AQP5, 1:50, Abcam, Cambridge, MA, USA) or rabbit anti-H_4_R (1:100) (Alpha Diagnostic International, San Antonio, TX, USA), antibodies overnight in a humidified chamber at 4^°^C. Immunoreactivity was detected by using horseradish peroxidase-conjugated anti-mouse or anti-rabbit antibodies and visualized by diamino-benzidine staining (Sigma Chemical Co., St. Louis, MO, USA). Specimens were counterstained with hematoxylin.

Apoptotic cells were detected, as earlier reported [3,5,7], using Apoptag^TM^ plus peroxidase in situ apoptosis Detection Kit (CHEMICON International, Temecula, CA, USA) according to the manufacturer’s instructions.

Light microscopy was performed on an Axiolab Karl Zeiss microscope (Göttingen, Germany). All photographs were taken using a Canon PowerShot G5 camera (Tokyo, Japan).

### Micronucleus assay

A micronucleus test performed in polychromatic erythrocytes of bone marrow was carried out according to Schmid [14] and also in peripheral blood, with minor modifications [15]. Bone marrow smears were prepared from rat femurs while peripheral blood samples were obtained from the caudal vein of the same adults specimens. At least 1,000 cells per rat were counted, and the data were obtained from 6 rats. Micronucleus evaluation in small intestine was performed according to Vanhauwaert et al. [16] with adaptation as it was previously described [7]. The criteria of the micronucleus in bone marrow follow Schmid [14]: i) the same staining as the main nucleus; ii) smaller than 1/3 diameter of the main nucleus; iii) not attached to the main nucleus. Cells were visualized and micronuclei were counted under Axiolab Karl Zeiss microscope (Göttingen, Germany).

### Salivary secretion

Salivation was assessed in anesthetized rats (chloralose 100 mg/kg, 0.5 ml NaCl-0.9%) (FLUKA, Berlin, Germany) as it was previously described [3]. Briefly, the right femoral vein was cannulated to administer the sialagogic agonist, methacholine (FLUKA) at different concentrations (0.3, 1, 3, 10 and 30 µg/kg, in saline). No basal salivation was observed from the glands. Salivary samples were collected and the quantity of saliva was determined by weighing. Results were expressed as mg of saliva per gland.

### Evaluation of total thiols, TBARS levels and catalase activity

The thiobarbituric acid reactive species (TBARS) assay is a well-established method for screening and monitoring lipid peroxidation. The method used in the present study, was previously described by Yagi [17]. Briefly, a portion of tissue was homogenized in phosphate buffer pH 7.4, and was transferred to a tube containing trichloroacetic acid, HCl and 2-thiobarbituric acid. The mixture was boiled for 1 h and then centrifuged at 3,000 rpm for 20 min at 4^°^C. The absorbance in the resulting pink-stained TBARS supernatants was determined in a spectrophotometer (Genesys 10 UV, Thermo Scientific) at 535 nm. The acid did not produce color when tested without the addition of the sample. A molar extinction coefficient of ε = 1.56 x 10^5^ M^-1^ cm^-1^was used for calculations.

Tissue total thiols concentration was estimated by the ability of the sulfhydryl group to reduce 5,5´-dithiobis(2-nitro-benzoic acid) (DTNB, Sigma-Aldrich, St. Louis, Mo, USA) according to Tietze [18]. Briefly, 35 mg of tissue were homogenized with ice-cold HClO_4_ (0.5 N) and centrifuged for 10 min at 600g. The pH of the supernatant was neutralized with Na _3_PO_4_. 20 µl of the supernatant were mixed with 50 µl of freshly prepared DTNB solution (6 mM) in 1 ml of 100 mM phosphate buffer and the mixture was incubated for 1 min to form a yellow-colored anionic product whose absorbance is measured at 412 nm against a reagent blank. A molar extinction coefficient of ε = 13.6mM^-1^ cm^-1^was used for calculations.

Catalase activity was measured spectrophotometrically by monitoring the disappearance of H_2_O_2_ at 240 nm, as it was previously described [8]. A unit of catalase was defined as the disappearance of 1 µmol of H_2_O_2_/min (ε 43.6 M^-1^cm^-1^).

### Cell culture and radiation dose–response curves

The human breast cancer cell lines MDA-MB-231 and MCF-7 (American Type Tissue Culture Collection, VA, USA) were cultured in supplemented RPMI 1640 as it was previously described [8]. For the radiosensitivity studies, MCF-7 and MDA-MB-231 cells were seeded in 6-well plates (1,200 cells/well) and were treated with 10 µM JNJ7777120 or remained untreated. The radiobiological parameters (SF 2Gy: fraction of surviving cells after exposure to 2 Gy dose; Dose 0.01: dose that reduces survival to 1%; Dose 0.10: dose that reduces survival to 10%) were calculated from the clonogenic surviving curves as it was described [8].

### Statistical analysis

Data shown are mean ± standard error of the mean (SEM) of at least two independent experiment. Statistical evaluations were made by analysis of variance (ANOVA) that was followed by Newman-Keuls’ Multiple Comparison Test, using GraphPad Prism Version 5.00 software (San Diego, CA, USA). P values < 0.05 were considered significant.

## Results

### Radioprotective effect of JNJ7777120 on small intestine

**Figure 1:**
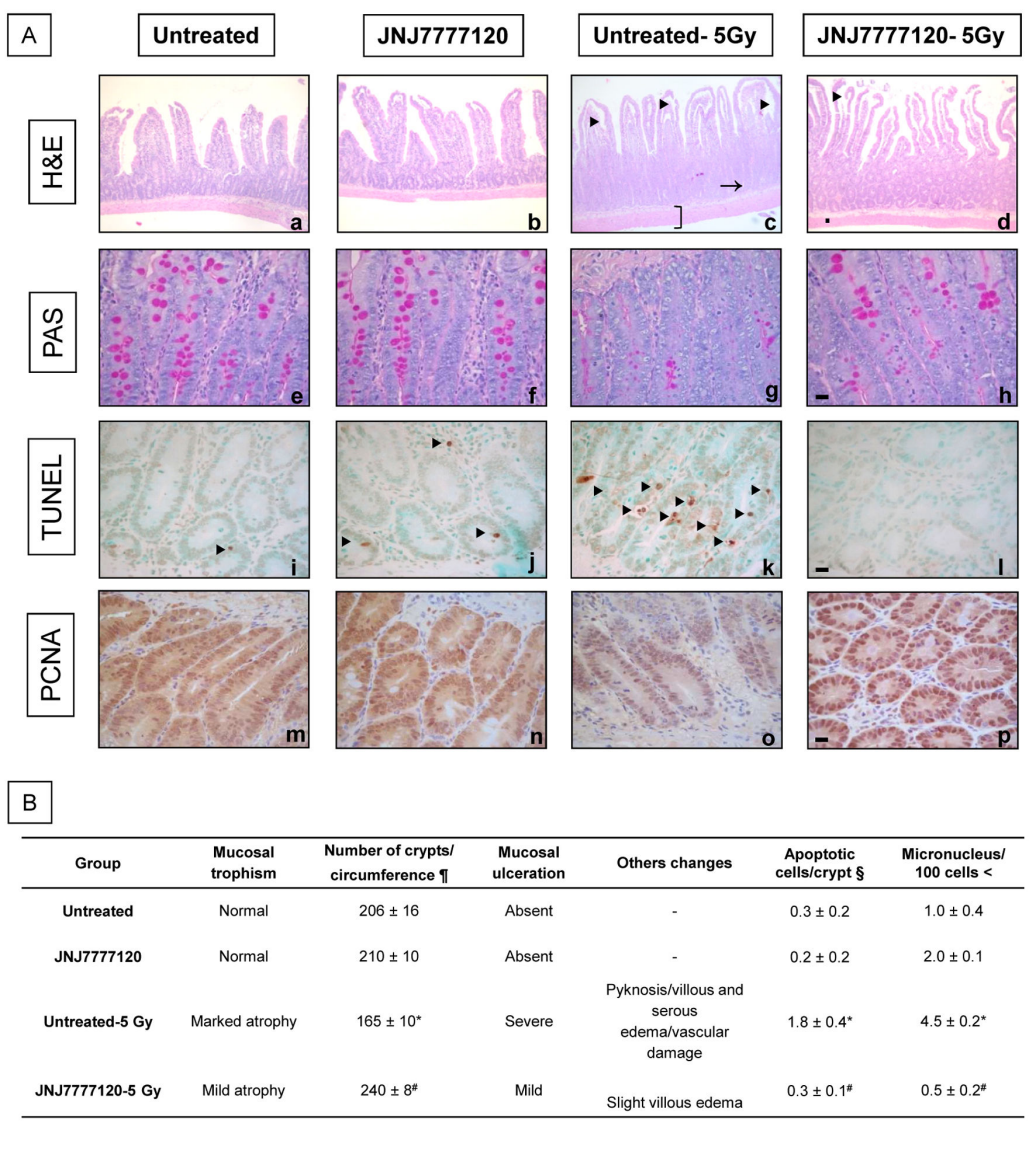
Effect of JNJ7777120 on radiation-induced cytotoxic and genotoxic damage of rat small intestine. (A) Representative pictures of (a,e) Normal histological appearance of untreated and (b,f) JNJ7777120-treated small intestine. (c,g) Intestine of irradiated rats displaying reduction in the number of crypts (arrow) with decreased goblet cells (g), villous edema (arrow head) and severe edema in serosa ([, c). (d,h) Intestine of JNJ7777120-treated and irradiated animals showing preservation of the mucosa, absence of vascular damage and an increased number of crypts (d) with restored presence of goblet cells (h). (a–d) H&E staining. (e–h) PAS staining. (i) Occasional TUNEL-positive cells in crypts in untreated and (j) JNJ777120-treated rats. (k) Massive presence of TUNEL-positive cells in crypts of irradiated rats. (l) Significant reduction of TUNEL-positive cells in crypts of treated and irradiated rats. Arrow head indicate positive cells. (m–p) Similar PCNA immunoreactivity in crypts from all groups. Pictures were taken at 630x-fold magnification. Scale bar= 20 µm. (B) Histopathological characteristics and average number of apoptotic cells and micronucleus in small intestine. §The number of TUNEL positive cells per crypt are expressed as means ± SEM. <The number of micronuclei per 100 cells are expressed as means ± SEM. *P<0.05 vs. Untreated; ^#^ P<0.05 vs. Untreated-5Gy.

We first evaluated the effect of JNJ7777120 in radiation-induced damage on small intestine. JNJ7777120 preserved histopathological characteristics of rat small intestine 3 days post-irradiation. JNJ7777120 diminished mucosal atrophy, disruption of muscular layer fibers, villous edema and preserved the number of crypts after radiation exposure (240±8 vs. 165±10, P<0.05). In addition to the preservation of mucosal trophism, JNJ7777120 also restored the radiation-induced reduction in goblet cells in crypts evaluated with PAS staining and prevented severe serous edema (Figure 1).

Furthermore, at 3 days post-irradiation a significant increase in pyknotic nuclei corresponding to apoptotic cells in crypt was observed, effect that was completely blocked by JNJ7777120 treatment. On the other hand, the marker of proliferation PCNA was high in both untreated and JNJ7777120-treated rats (Figure 1) *.*

Micronuclei assay was investigated as a biomarker for evaluating ionizing radiation-induced chromosomal damage. The number of micronuclei in intestinal cells was significantly increased after irradiation, and JNJ7777120 treatment normalized it (0.5±0.2 vs. 4.5±0.2, P<0.05) (Figure 1) *.*

Histopathological changes of small intestine were associated with an increase in TBARS and a decrease in total cellular thiols, while no significant changes of catalase activity was observed (Table 2). Significant improvement of TBARS and thiol levels was observed after JNJ7777120 treatment (Table 2).

**Table 2:** Effect of JNJ7777120 on thiobarbituric acid reactive substances (TBARS) level, total thiols content and catalase activity in small intestine, spleen and submandibular glands (SMG) of irradiated and non-irradiated rats.

	**Groups**			
	**Untreated**	**JNJ7777120**	**Untreated- 5Gy**	**JNJ7777120- 5Gy**
**Intestine**				
**TBARS**	0.40±0.02	0.33±0.04	0.78±0.02*	0.23±0.03#
**Total Thiols**	4.78±0.20	6.00±0.40	3.83±0.38	4.75±0.29
**Catalase Activity**	7.83±1.08	6.65±0.52	5.95±0.69	5.91±0.71
**Spleen**				
**TBARS**	0.18±0.02	0.15±0.01	0.35±0.05*	0.16±0.02#
**Total Thiols**	4.14±0.42	3.66±0.02	4.36±0.60	4.14±0.70
**Catalase Activity**	780.00±55.80	692.30±67.30	750.30±42.20	1416.00±55.10*#
**SMG**				
**TBARS**	0.18±0.01	0.19±0.02	0.17±0.01	0.16±0.01
**Total Thiols**	3.76±0.31	3.04±0.20	2.36±0.11*	2.44±0.26*
**Catalase Activity**	31.88±2.14	28.80±0.97	43.48±1.81*	49.46±1.91*

Ionizing radiation augmented TBARS level in the intestine and spleen compared to untreated rats while JNJ7777120 blocked this effect. Data represent the means ± SEM (* P<0.05 vs. Untreated; # P<0.05 vs. Untreated-5Gy). (TBARS are expressed as nmol/mg protein.) Total thiols decreased in SMG of untreated-5Gy and JNJ7777120-5Gy rats compared to untreated rats. Data represent the means ± SEM (* P<0.05 vs. Untreated). (total thiols are expressed as mmol/mg tissue.) Catalase activity augmented in the spleen of JNJ7777120-5Gy rats and in the SMG of untreated-5Gy and JNJ7777120-5Gy rats. Data represent the means ± SEM (* P<0.05 vs. Untreated; # P<0.05 vs. Untreated-5Gy). (catalase activity is expressed as U/mg protein.)

### Effect of JNJ7777120 on SMG of irradiated animals

**Figure 2:**
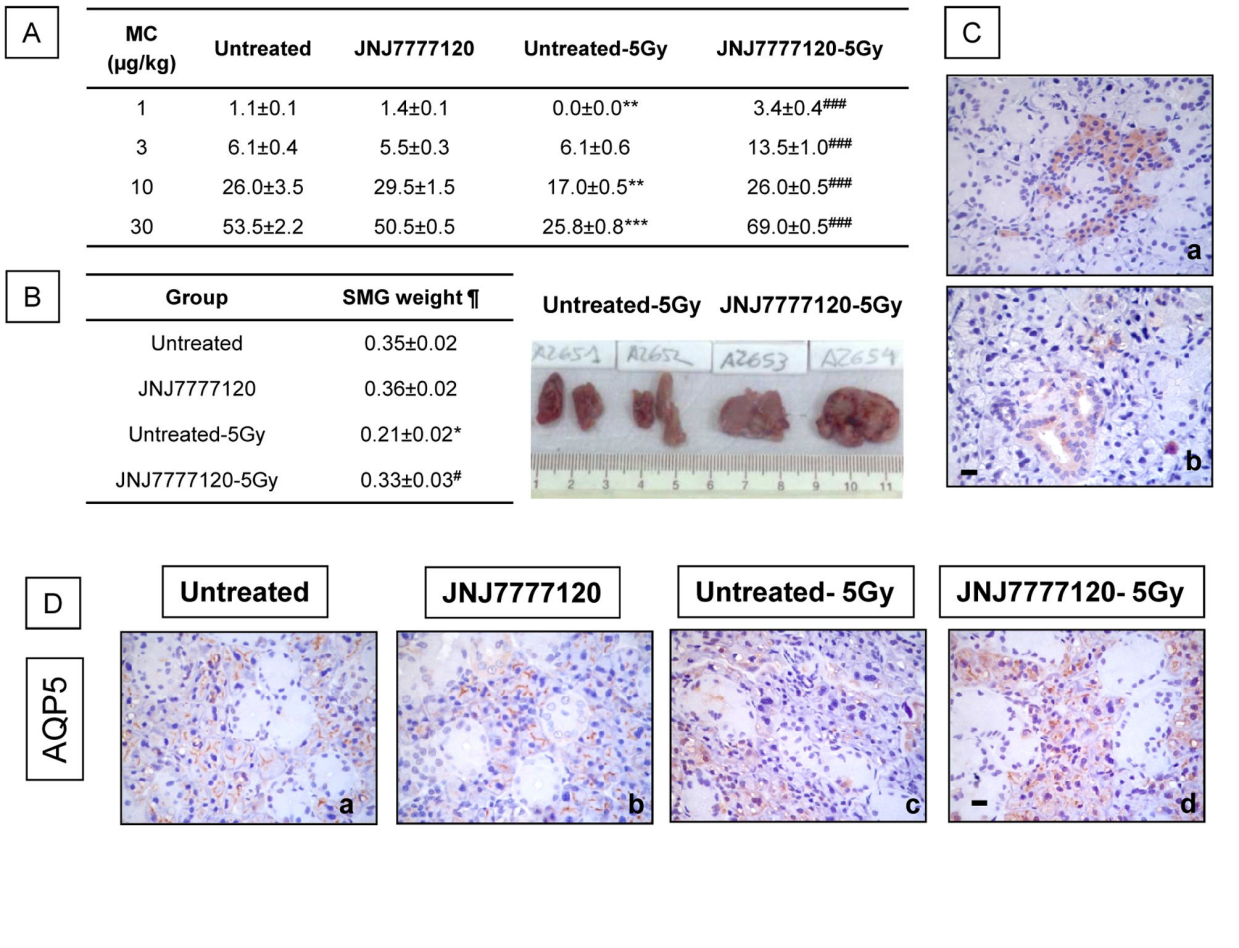
Effect of JNJ7777120 on radiation-induced damage on salivary function. (A) Mean salivary secretion in irradiated and non-irradiated, untreated and JNJ7777120-treated rats. Error bars represent the means ± SEM (**P<0.01, ***P<0.001, vs. Untreated, ###P<0.001 vs. Untreated-5Gy). (B) ¶SMG’s percentage of body weight (SMG weights were divided by total body weight in grams and multiplied by 100). Data represent the means ± SEM (*P<0.05 vs. Untreated; #P<0.05 vs. Untreated-5Gy). Inset: JNJ7777120 compound significantly preserves SMG mass. (C) H_4_R immunoreactivity. H_4_R was detected in some (a) acini and also (b) excretory ducts. (D) AQP5 immunoreactivity. AQP5 was detected almost exclusively in acini in (a) untreated, (b) JNJ777120-treated and (d) treated and irradiated rats. (c) Reduce AQP5 immunoreactivity and altered distribution in SMG of irradiated rats. Pictures were taken at 630x-fold magnification. Scale bar= 20 µm.

We further investigated the effect of JNJ7777120 in radiation-induced functional and morphological alterations of SMG. SMG function was evaluated by determining the salivation after methacholine stimulation in treated and untreated animals. Radiation significantly reduced salivary secretion by approximately 35-50% at 10 and 30 µg/Kg methacholine concentration in comparison to untreated rats while decreased SMG wet weight relative to body weight (Figure 2 A & B). On the other hand, JNJ7777120 treatment completely blocked radiation-induced reduced salivation at all concentration of methacholine used and this was associated with a conservation of SMG wet weight (Figure 2 A & B).

**Figure 3:**
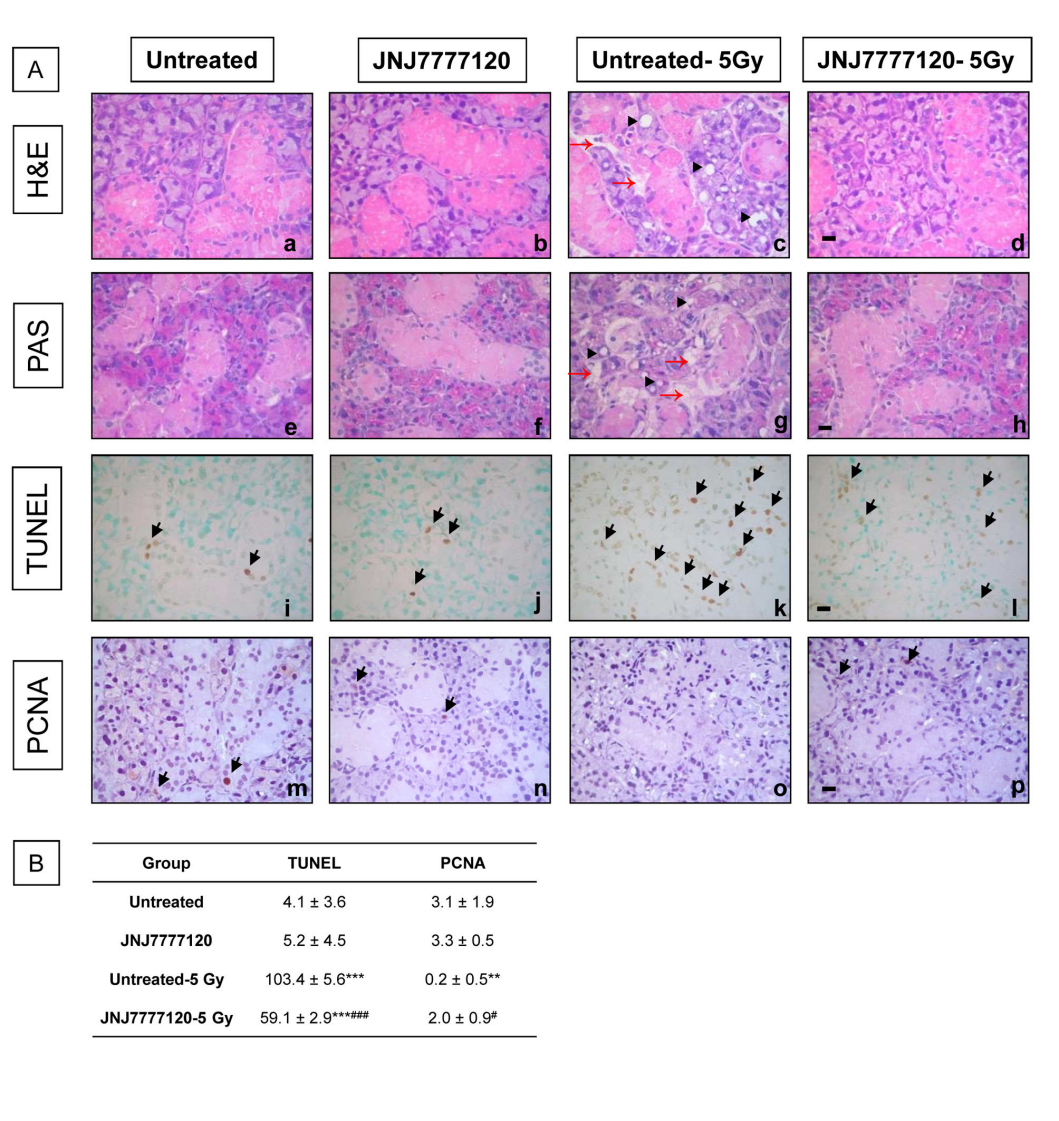
Effect of JNJ7777120 on radiation-induced morphological, proliferative and apoptotic alterations in the rat SMG. (A) SMG histopathology. (a,e) Normal histological appearance of untreated and (b,f) JNJ7777120-treated SMG (c, g). SMG of irradiated rats displaying damage in the epithelial architecture of the granular convoluted ducts, mild edema (red arrow), partial loss of eosinophilic secretor granular material and vacuoles (arrow head). (d,h) SMG of JNJ7777120-treated and irradiated animals showing preserved structure organization of secretor granules, with normal appearance of granular convoluted ducts with eosinophilic secretion. (a–d) H&E staining. (e–h) PAS staining. (i) Occasional TUNEL-positive cells in glandular duct cells in untreated and (j) JNJ777120-treated rats. (k) Massive presence of TUNEL-positive cells in ductal and acinar cells of glands of irradiated rats. (l) Significant reduction of TUNEL-positive cells in glands of treated and irradiated rats. (m,n) Similar PCNA immunoreactivity in SMG from untreated and JNJ7777120-treated rats. (o) Almost total absence of PCNA immunoreactivity in irradiated gland. (p) Partial preservation of PCNA-positive cells in treated and irradiated glands. Arrows indicate positive cells. Pictures were taken at 630x-fold magnification. Scale bar= 20 µm. (B) Average number of apoptotic cells and PCNA-positive cells are shown. Error bars represent the means ± SEM. **P<0.01, ***P<0.001 vs. Untreated; ^#^ P<0.05, ^# # #^ P<0.001 vs. Untreated-5Gy.

Histopathological analysis demonstrated that radiation produced severe gland atrophy with alteration in the epithelial architecture, partial loss of eosinophilic secretor granular material, increased acinar vacuolization and mild edema. JNJ7777120 treatment completely prevented histological damage of SMG, which showed normal histological features (Figure 3 A).

It is important to note that in non-irradiated rats, non-significant effects were produced by JNJ7777120 in functional or morphological characteristics of SMG (Figures 2 & 3).

In addition, results show that the water channel protein aquaporin-5 (AQP5) was localized mainly in the apical membranes of acinar cells of non-irradiated SMG, and treatment with JNJ7777120 did not affect AQP5 distribution. Radiation diminished AQP5 immunoreactivity and altered its localization, which was observed at the apical, basolateral, and basal plasma membrane. JNJ7777120 seemed to preserve AQP5 immunoreactivity and distribution in irradiated SMG (Figure 2 D).

Furthermore, it has been suggested that the radiation-induced imbalance between apoptosis and proliferation in SMG is responsible for the impairment of salivary gland function that leads to a decreased salivation [3]. Therefore, the JNJ7777120-mediated modulation of proliferation (PCNA) and apoptosis (TUNEL) markers were also evaluated by immunohistochemical analysis.

Figure 3 shows a very low PCNA expression and a small number of TUNEL-positive cells, which were not modified by JNJ7777120 treatment in non-irradiated rats. Irradiation markedly reduced PCNA immunoreactivity while significantly increased apoptotic cells in all gland compartments including acinar cells, granular convoluted cells, intercalated ductal cells and excretory ducts. In contrast, JNJ7777120 administration produced a partial preservation of PCNA-positive cells while reduced the number of apoptotic cells in the SMG of irradiated animals (59.1±2.9 vs. 103.4±5.6, P<0.001).

In addition, the immunohistochemical analysis of H_4_R showed that it was detected in some acini and also ducts of rat SMG (Figure 2 C).

Thiol levels were decreased and catalase activity was increased, while TBARS levels were not modified upon radiation exposure and JNJ7777120 administration was unable to modify these parameters (Table 2).

### Effect of JNJ7777120 on hematopoietic tissue of irradiated animals

**Figure 4:**
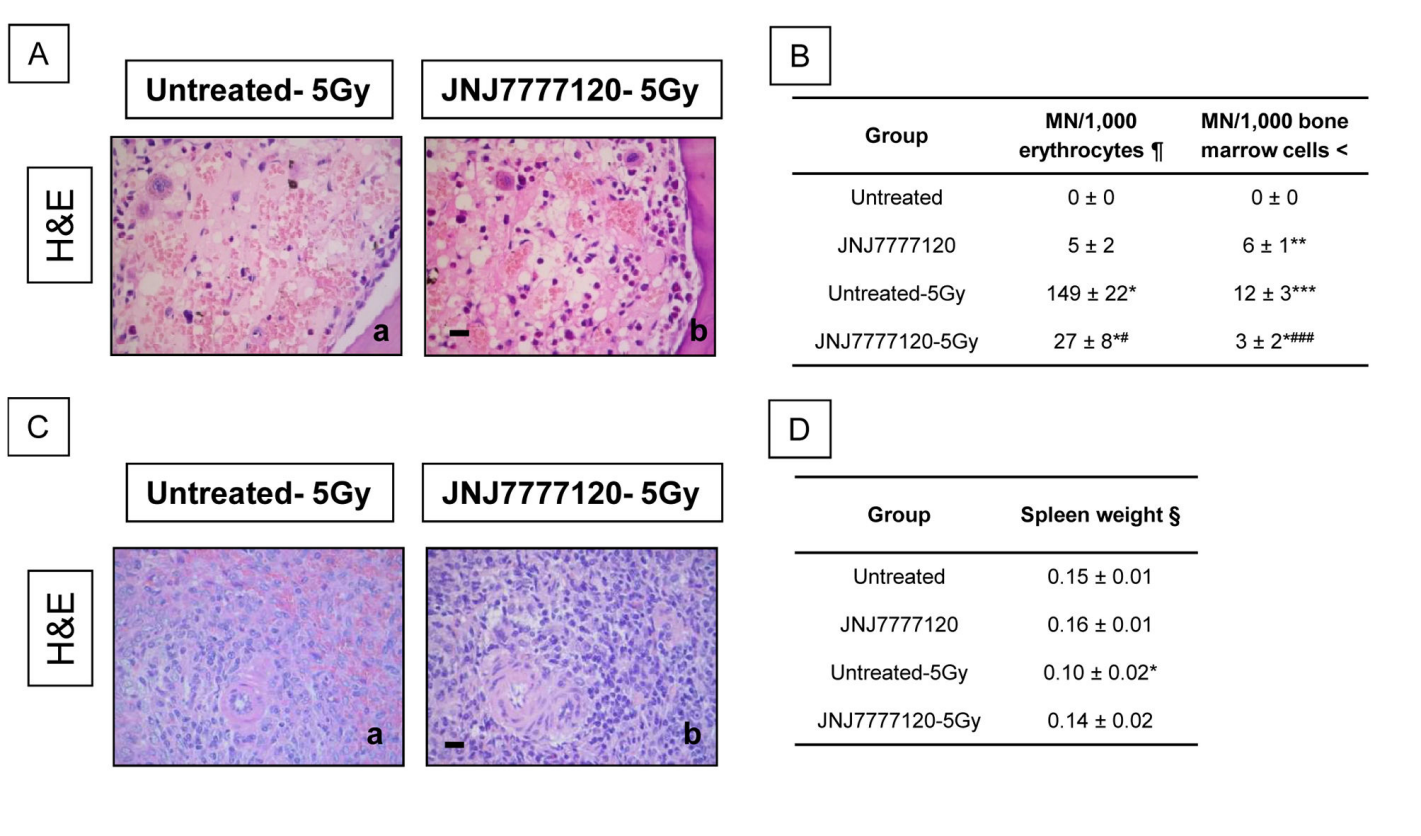
Evidence of the radioprotective effect of JNJ7777120 on rat hematopoietic tissue 3 days after irradiation. (A) H&E stained representative bone marrow sections of (a) untreated-5Gy and (b) JNJ7777120-5Gy rats. Pictures were taken at 630x-fold magnification. Scale bar= 20 µm. (B) Micronucleus frequency. ¶ The number of micronuclei (MN) was determined in 1,000 erythrocytes and is expressed as mean ± SEM. < The number of micronuclei (MN) was determined in 1,000 bone marrow cells and is expressed as mean ± SEM (*P<0.05, **P<0.01, ***P<0.001 vs. Untreated; #P<0.05, # # #P<0.001 vs. Untreated-5Gy). (C) H&E stained representative spleen sections of (a) untreated-5Gy and (b) JNJ7777120-5Gy rats. Pictures were taken at 630x-fold magnification. Scale bar= 20 µm. (D) § Spleen’s percentage of body weight (spleen weights were divided by total body weight in grams and multiplied by 100). Data represent the means ± SEM (*P<0.05 vs. Untreated).

Pathological examinations of bone marrow 3 days after total body irradiation show that JNJ7777120 administration efficiently ameliorated the radiation-induced reduction of bone marrow cells (aplasia) (Figure 4 A). This effect was correlated with a decrease in the radiation-induced enhanced micronuclei production in bone marrow cells (Figure 4B). In agreement with these results, JNJ7777120 treatment reduced micronuclei frequency in peripheral blood (27±8 vs. 149±22, MN/1,000 erythrocytes, P<0.05) (Figure 4 B).

**Figure 5:**
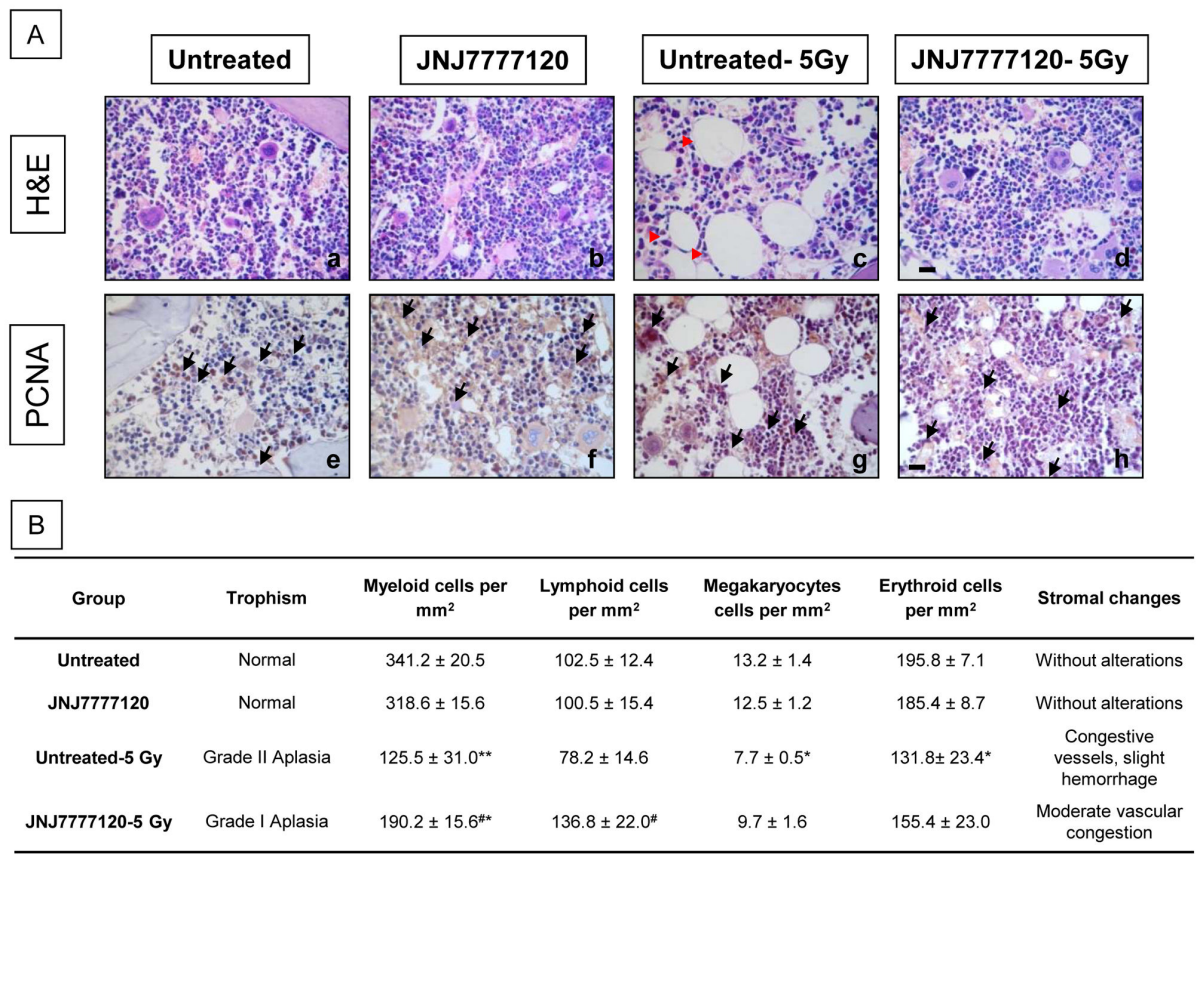
Effect of JNJ7777120 on bone marrow repopulation 30 days after whole body irradiation. (A) Bone marrow histopathology. (a,e) Normal trophism of untreated, and (b,f) JNJ7777120-treated bone marrow. (c,g) Bone marrow of irradiated rats showing a reduced number of components and an important adipose replacement (red arrow head). (d,h) Bone marrow of treated and irradiated animals demonstrating significant preservation of bone marrow components and minor adipose replacement. (a–d) H&E staining. (e–h) PCNA immunoreactivity. Arrows indicate PCNA-positive cells. 630x-fold magnification. Scale bar= 20 µm. (B) Histopathological characteristics of rat bone marrow. Error bars represent the means ± SEM (*P<0.05, **P<0.001 vs. Untreated; ^#^ P<0.05 vs. Untreated-5Gy).

Accordingly, JNJ7777120 enhanced bone marrow repopulation after 30 days of radiation exposure. JNJ7777120 almost completely conserved the medullar progenies, preserving myeloid, erythroid, lymphoid and megakaryocytic precursors while diminished the adipose replacement compared to bone marrow derived from untreated and irradiated rat group (Figure 5). In addition, similar PCNA immunoreactivity was observed in bone marrow from the different experimental groups (Figure 5).

Furthermore, radiation reduced the number of germinal centers, considerably decreased cellularity of immunocompetent cells, and enhanced vascular damage with focal hemorrhage in spleen. JNJ7777120 treatment significantly preserved spleen cellularity and germinal centers to almost normal extent (Figure 4 C). The preservation of spleen histology produced by JNJ7777120 was associated with a conservation of spleen wet weight, which was reduced after radiation exposure (Figure 4 D). Also, a significant increase in TBARS levels was observed after radiation exposure, while JNJ7777120 treatment completely reversed this effect and increased catalase activity in spleen. Total thiols were not significantly modified in irradiated and non-irradiated, treated or untreated spleen (Table 2).

### Effect of JNJ7777120 on the radiobiological parameters of breast cancer cells

**Figure 6:**
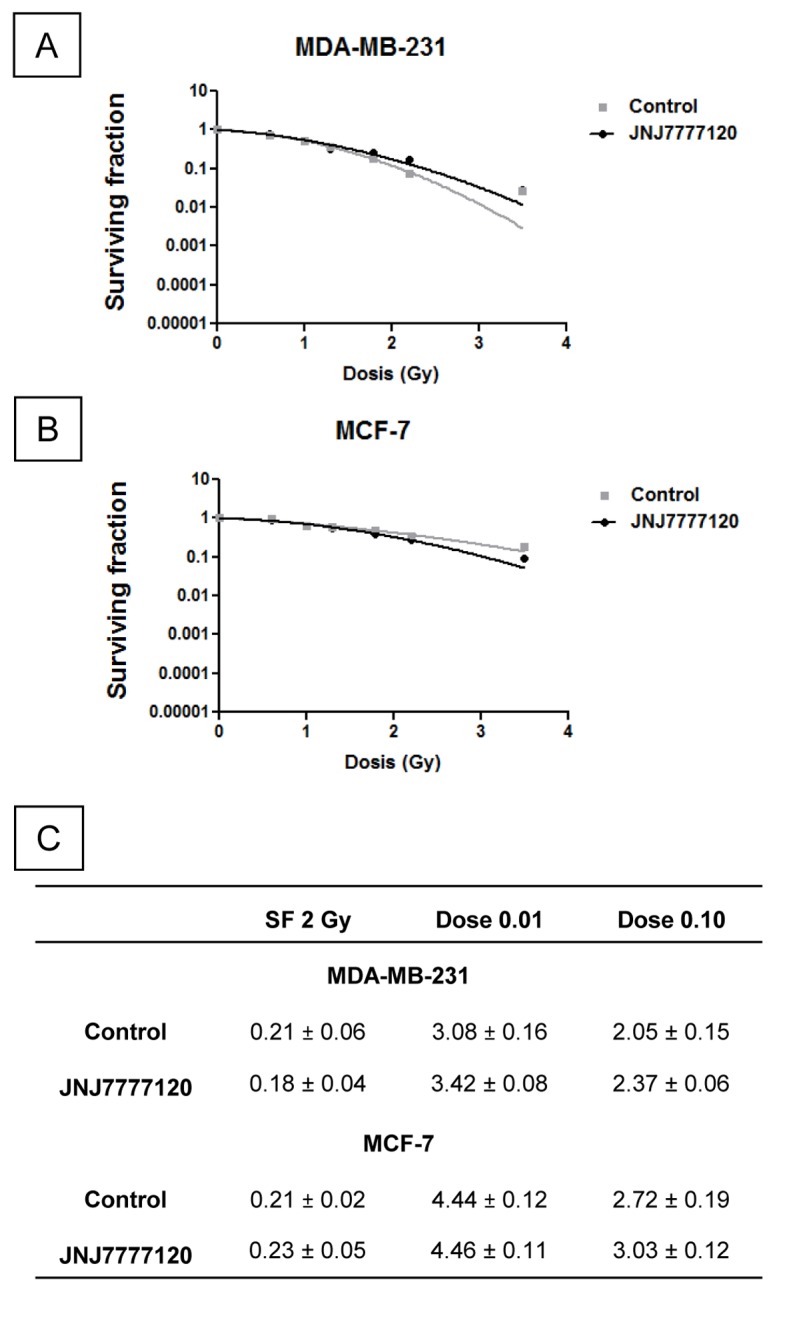
Effect of JNJ7777120 on the radiobiological parameters of two human breast cancer cell lines. (A) MDA-MB-231 and (B) MCF-7 cells were cultured in presence or absence of 10 µM JNJ7777120 and clonogenic survival was determined. (C) Radiobiological parameters (SF 2Gy, Dose 0.01, Dose 0.10) were obtained from the survival curves adjusted to the linear quadratic model [SF=e^-(αD+βD2)^]. Values are means ± SEM.

In order to determine the JNJ7777120 compound selective cytoprotection of normal tissues, we also evaluated the radiobiological response of two human breast cancer cells. Results indicate that the radiobiological parameters of MCF-7 and MDA-MB-231 cells were not modified upon JNJ7777120 treatment (Figure 6).

## Discussion

This study aims to investigate the radioprotective effect of JNJ7777120 compound on gamma radiation-induced damage on small intestine, SMG and hematopoietic tissue.

The microcolony assay, developed by Withers and Elkind, measures the number of regenerating crypts per intestinal circumference at 3-4 days after radiation, serves as a surrogate for stem cell survival, directly quantifies radiation dose-dependent lethality of the crypts and is a predictive measure of gastrointestinal damage because it is not influenced by concomitant damage to other organs such as bone marrow [1].

Whole body irradiation produced epithelial cell damage in small intestine, which accounts for reduction of the number of regenerating crypts, structural changes of the villi, vascular damage and mucosal superficial ulceration as it was previously described [7]. JNJ7777120 treatment was effective in preventing radiation-induced damage to small intestine, preserving mucosal epithelium, with absence of vascular damage and an increased number of crypts that were comparable to non-irradiated animals.

The gastrointestinal tract is covered by a lubricant mucous layer that constitutes a protective barrier against microorganisms, physical and chemical attacks. Mucus is secreted by goblet cell, which arise from pluripotent stem cells present at the base of crypt [19]. Goblet cell ablation has been recognized as one of the most consistent hallmarks of chemotherapy- and radiotherapy-induced intestinal damage [20]. Irradiation decreases the number of goblet cells in crypts, resulting in a reduction of the production of mucin while JNJ7777120 restores the number of goblet cells. Previous acute radiation data indicate that the response of goblet cell is complex, decreasing in cell number at 72 h after irradiation, while others demonstrate increases particularly after several weeks and months [19].

Radiation induces apoptosis of radiosensitive cells in the intestinal crypt cells, which are responsible for renovation and maintenance of the intestinal cellular architecture [1]. The protective effect of JNJ7777120 on small intestine was mainly associated with a reduced apoptosis in intestinal crypts of irradiated rats.

It is well known that the biological effects of ionizing radiation results principally from damage to DNA and double-strand breaks, which can lead to chromosomal aberrations and are the most relevant lesions responsible for most biologic insults, including cell killing [1]. The micronucleus test is a tool for genotoxic assessment, being a reliable biomarker for evaluating ionizing radiation-induced chromosomal damage [7,21]. JNJ7777120 administration reverses radiation-induced micronuclei frequency in intestinal cells, suggesting that this compound reduced radiation-induced genotoxic damage.

Similar radioprotective effects were observed on rat and mouse small intestine upon histamine administration (0.1 mg/kg) [5,7].

Irradiation is a central treatment modality administered for head and neck cancers. However, a significant drawback of radiotherapy is the severe and irreversible gland functional and structural alterations, which result in salivary dysfunction and consequent xerostomia [1,22]. Present findings demonstrate that JNJ7777120 administration can significantly protect the SMG from deleterious effects of irradiation. Histological studies revealed radiation-induced prominent atrophy and degeneration. JNJ7777120 completely reverses radiation-induced reduced salivary secretion and histological alterations, preserving SMG function. The data indicate that the cytoprotective mechanisms of JNJ7777120 may be related to improved cell proliferation and inhibition of cell apoptosis.

The water channel protein AQP5 is highly expressed in salivary glands and is important for salivary secretion, considering that knockout mice lacking AQP5 show markedly depressed rates of salivation. AQP5 is localized almost exclusively at the apical plasma membrane of the acinar and the intercalated duct cells in the rat SMG [23]. Ionizing radiation significantly down-regulates AQP5 expression in the SMG, which may contribute to radiation-induced salivary dysfunction[24,25]. . Present results show that AQP5 is localized in the apical membranes of acinar cells of non-irradiated SMG, while radiation diminished AQP5 immunoreactivity and altered its localization, which was not principally observed at the apical plasma membrane. JNJ7777120 administration seemed to preserve AQP5 distribution, suggesting that the protection of acinar cells by JNJ7777120 might also involve conservation of AQP5 expression with typical localization. In this sense, other experimental radioprotective molecules reduce radiation-induced salivary gland damage by preventing the reduction of AQP5 expression [25].

The presence of H_1_R and H_2_R in salivary glands was previously described [26]. In this study, we demonstrated the expression of H_4_R in some acinar and excretory ductal cells by immunohistochemistry, indicating that JNJ7777120 could be acting on H_4_R expressed in SMG. In line with our results, it was recently reported the expression of H_4_R in human salivary glands. H_4_R level was reduced in salivary glands of patients with Sjögren’s syndrome, which is a chronic autoimmune disease characterized by severe alterations in both the quality and quantity of saliva and tears [11,27], suggesting that H_4_R may play some constitutive role in the maintenance of healthy salivary epithelium and salivation. Also, comparable SMG radioprotection was observed with histamine treatment (0.1 mg/kg) [3].

Whole-body radiation can cause severe damage to the hematopoietic system, causing immediate cell death and mitotic inhibition that lead to depopulation of bone marrow. As a result, replenishment of peripheral blood does not take place, leading to anemia and leucocytopenia, and therefore it is necessary to identify a novel strategy for overcoming this injury [1,2]. Results show that JNJ7777120 administration relieves bone marrow aplasia and decreases occurrence of bone marrow and peripheral blood micronucleated erythrocytes 3 days after radiation, indicating that JNJ7777120 administration could reduce the genotoxic effect of total body irradiation in the hematopoietic system. Also, JNJ7777120 markedly improves the repopulation 30 days post-irradiation, accelerating hematopoietic recovery.

Under pathological conditions, hematopoiesis may emigrate from these bone marrow sites and may continue its cell production much less efficiently, in other extramedullary sites, such as spleen [1]. Administration of JNJ7777120 before irradiation significantly prevents radiation-induced decrease in wet weight and histopathological alterations in the spleen.

Our data obtained from *in vivo* experiments provide strong evidence that JNJ7777120 efficiently protects normal tissues against ionizing radiation-induced toxicity. JNJ777120 clearly preserves the major histological parameters of the small intestine and salivary gland, conserving intestinal crypts and SMG functionality, while relieves radiation-induced myelosupression and accelerates bone marrow recovery. The reduction of radiation-induced apoptosis and genotoxic damage are to be likely mechanisms for the radioprotective activity of JNJ7777120.

In addition, radiation damage is to a large extent, caused by the overproduction of ROS, which overwhelm the levels of antioxidants, resulting in oxidative stress that not only damage DNA but also cellular membranes, leading to lipid peroxidation [28]. A significant increase in lipid peroxidation as measured by TBARS, was observed 3 days after whole body irradiation in spleen and small intestine while its level showed no significant variation in SMG. Administration of JNJ7777120 completely reversed this oxidative damage induced by radiation in both tissues, suggesting that it could protect cellular membrane from radiation-induced lipid peroxidation. The radioprotective effect of this compound was not associated with a modulation of total cellular thiols. According to the data obtained, other potential radioprotectors reduce oxidative damage [2], however further studies are needed to fully characterized the antioxidant status in whole body irradiated and JNJ7777120-treated rats.

In order to consider JNJ7777120 as a selective radioprotector and therefore a clinically relevant agent able to protect normal tissues without protecting tumoral cells, the potential protection of tumor cells has to be evaluated. Present findings demonstrate that JNJ7777120 was not able to modulate significantly the radiosensitivity of two H_4_R expressing human breast cancer cells [8,29], suggesting that it does not interfere with the anti-tumor efficacy of radiation. Moreover, this compound does not modulate growth of melanoma and pancreatic carcinoma cell lines [30].

Hematopoietic tissue, small intestine and salivary glands express H_4_R, which has become a major target of novel therapeutics. The discovery of JNJ7777120 as the first orally active, potent and selective non-imidazole H_4_R antagonist allowed a significant advance in the understanding of the pharmacology and function of H_4_R both *in vitro* and *in vivo* [10]. Paradoxically, histamine (0.1 mg/kg) treatment produces similar radioprotective effects on small intestine, bone marrow and SMG [3,5–7], suggesting that JNJ7777120 could exhibit agonistic activity in this native biological system. These results are consistent with recent evidence, which show that JNJ7777120 cannot be considered the "standard" H_4_R antagonist anymore, but depending on the parameter assessed, JNJ7777120 may also act as agonist to human H_4_R. It is noteworthy that, with respect to β-arrestin recruitment that can occur independently of G protein activation, JNJ7777120 acts only as partial agonist, but with respect to ERK activation, JNJ7777120 is actually a full agonist. Additionally, at mouse, rat and dog H_4_R, histamine is a full agonist with respect to Gi activation in Sf9 insect cell membranes, whereas JNJ7777120 is a partial agonist at these H_4_R orthologs. These data indicate that the H_4_R joins the growing family of 7 trans-membrane receptors showing functional selectivity or biased signaling [12,13]. Therefore, our results will also contribute to a better understanding of the pathophysiological function of the H_4_R in a native non-recombinant system.

In addition, it is important to highlight that side effects were not observed in most important organs including bone marrow and liver upon JNJ7777120 administration (data not shown).

In conclusion, these findings clearly confirm that JNJ7777120 exhibits protective effects on the small intestine, salivary glands and the hematopoietic system against whole-body gamma-radiation. For that reason, JNJ7777120 is a promising radioprotective agent with potential application in radiation protection for cancer patients who receive radiotherapy or radiosurgery.
